# Cross-Cultural Adaptation of the Functional Assessment of Cancer Therapy-Breast (FACT-B) in Malaysian Breast Cancer Survivors

**DOI:** 10.31557/APJCP.2021.22.4.1055

**Published:** 2021-04

**Authors:** Khairunnisa’ Md Yusof, Rozi Mahmud, Maha Abdullah, Kelly A. Avery-Kiejda, Rozita Rosli

**Affiliations:** 1 *Department of Biomedical Sciences, Faculty of Medicine and Health Sciences, Universiti Putra Malaysia, Serdang, Selangor, Malaysia. *; 2 *Priority Research Centre for Cancer Research, Innovation and Translation, School of Biomedical Sciences and Pharmacy, Faculty of Health and Medicine, University of Newcastle, NSW, Australia. *; 3 *Centre for Diagnostic Nuclear Imaging, Universiti Putra Malaysia, Serdang, Selangor, Malaysia. *; 4 *Department of Pathology, Faculty of Medicine and Health Sciences, Universiti Putra Malaysia, Serdang, Selangor, Malaysia. *

**Keywords:** QOL, breast cancer, FACT-B, Malay version, validity

## Abstract

**Introduction:**

The survival rate of female breast cancer survivors has been reported to be higher than other types of cancer in Malaysia. Nonetheless, breast cancer survivors face new challenges from unwanted side effects of treatment or management such as fatigue, psychological disturbance, or arm swelling, which can lead to the decline of quality of life (QOL). This study aims to adapt the Malay version of the Functional Assessment of Cancer Therapy-Breast (FACT-B) to evaluate the QOL and to test its reliability and validity in Malaysian breast cancer survivors.

**Methods:**

The Malay version of the FACT-B, with Disabilities of Arms, Shoulders and Hands (DASH), and Patient Health Questionnaire Anxiety-Depression Scale (PHQ-ADS) were distributed to female breast cancer survivors which were recruited on a voluntary basis, from cancer support groups based in selected states in Malaysia. Reliability was assessed based on internal consistency (Cronbach’s α), whereas concurrent validity was examined by comparing domains in FACT-B with DASH and PHQ-ADS. Finally, total scores of each domain were analysed between lymphedema and without lymphedema groups for known-group validity.

**Results:**

A total of 113 breast cancer survivors agreed to participate (response rate = 100%) in the study. Our results showed that the Cronbach’s α value for Malay FACT-B is 0.88, and each domain ranged from 0.62 to 0.88. A strong correlation was found between the physical well-being domain of FACT-B with DASH. Meanwhile, the breast cancer scale (BCS) displayed significant correlation with the instrument, Patient Health Questionnaire- Anxiety Depression Scale (PHQ-ADS), indicating that multiple factors including psychological distress were measured in the BCS domain. Furthermore, the instrument was able to detect differences in physical, functional and QOL between participants from lymphedema and without lymphedema groups.

**Conclusion:**

The Malay version of the FACT-B demonstrated reliable properties and is effective in assessing QOL and can be applied in Malaysian breast cancer survivors.

## Introduction

The increasing trend of breast cancer incidence is an alarming sign for health problem in most Asian countries. Despite the increasing number of cases every year, breast cancer has been reported to have the highest survival rate, due to early detection and better disease prognosis. According to the National Cancer Registry of Malaysia (NCRM, 2018), the 5-year relative survival of female breast cancer is the fourth highest (66.8%), after thyroid (82.3%), prostate (73.0), and corpus uteri cancer (70.6%). Although the risk factors of breast cancer in Asian populations are similar to those in Western populations (Ahn et al., 2009), the prognostic factors may be different due to the influence of physical activity, dietary intake and lifestyle, which subsequently can affect the survivorship and quality of life (QOL) of breast cancer survivors (Islam et al., 2015). Therefore, assessment of QOL in breast cancer survivors is important to establish the association between lifestyle and overall survival after breast cancer diagnosis.

The first Functional Assessment of Cancer Therapy-Breast or FACT-B was developed by Brady et al., (1997) from FACT-General (FACT-G) by adding breast cancer scale (BCS) to assess multidimensional QOL in breast cancer patients. It consists of 37 questions, divided into five domains, namely functional well-being (FWB), social well-being (SWB), emotional well-being (EWB), physical well-being (PWB) and additional concern, BCS. FACT-B has been widely used in clinical studies across the world including the USA, European countries, Middle East, and Asian regions. The FACT-B has been translated into 50 different languages but validation studies of different versions are only reported in Chinese (Cheung et al., 2014), Korean (Yoo et al., 2005), Malayalam (Pandey et al., 2002), Spanish (Martinez et al., 2011), Arabic (Kobeissi et al., 2014) and Persian (Patoo et al., 2015). The validity study of the FACT-B in different versions is important considering that English is not the native language for majority of the countries including Malaysia. Given that not all citizens in Malaysia are proficient in English, adaptation of the FACT-B in the Malay version can reduce the bias on the interpretation, direct translation, and perception of the items among the participants (Arafat et al., 2016). Malay or *bahasa Melayu* is the main language in Malaysia and has been widely used for daily communication, official businesses and documentation by major ethnics in Malaysia, namely Malay, Chinese and Indians (Shamsudin et al., 2018). Therefore, evaluation and validation of the Malay version of FACT-B is important to ensure it is culturally relevant to be used in Malaysian breast cancer population and comprehensible while maintaining the meaning and content of the original items. 

## Materials and Methods

*Study subject recruitment*

A total of 113 breast cancer survivors were recruited from various cancer support groups from selected states in Malaysia. The sample size was estimated by applying subject to item ratio of 3:1 using the Sample Size Calculator (Arifin, 2018) to maintain a minimally acceptable Cronbach α = 0.65 (expected value = 0.8), significance level (α = 0.05) and power of 80% with expected dropout rate = 10%. The inclusion criteria for the subject selections were female, Malaysian citizen, aged above 18 years old, diagnosed with breast cancer, had undergone breast cancer surgery (mastectomy or lumpectomy), completed all treatments at least three months before recruitment, and no evidence of newly diagnosed cancer. A minimal cash incentive was given to each subject as a token for volunteering in the study. Informed consent was obtained from all participants before they were asked to self-administer the questionnaire. Ethics approval was obtained from The Ethics Committee for Research Involving Human Subjects Universiti Putra Malaysia (JKEUPM (IBS-P129) 2017) and was conducted according to the Declaration of Helsinki.

*Instrument*

The data on demographic (i.e., age, marital status, number of children, occupation, income and educational level) and medical history (age of diagnosis, date of breast cancer surgery, stage of breast cancer, types of breast surgery, and types of treatment) were collected from the self-administered questionnaire. The Malay version of FACT-B was obtained from FACIT.org with a license issued to the investigator. Considering that the questionnaire comprises short and straight forward questions, it was mainly self-administered, and interviews were only carried out with subjects who had problems in reading. The scoring method of FACT-B is in Likert’s scale, ranging from 0 to 4, where 0 represents ‘not at all’, 1 - a little bit, 2 - somewhat, 3 - quite a bit and 4 - very much. Total scores from each domain (PWB+SWB+FWB+SWB+BCS) were calculated based on instructions in the manual and classified as FACT-B total scores, with additional analysis of FACT-G (PWB+EWB+FWB+SWB) and trial outcome index (TOI), which summarised total scores of PWB+FWB+BCS. A final score of FACT-B was calculated by reversing some of the items (GP1 to GP7, GE1, GE3-GE6, B1-B3, B5-B8 and B10) as suggested in the manual (Brady et al., 1997). 

The validated Malay version of DASH (Al-Husuny, 2011) and psychometry instrument, Patient Health Questionnaire-Anxiety Depression Scale (PHQ-ADS) (Sherina et al., 2012; Sidik et al., 2012) questionnaires were also administered together with FACT-B for concurrent validity. The DASH questionnaire is an established instrument to assess the upper extremities function of the surgery side (left or right) which comprises 30 items that evaluate the ability of an individual to perform physical activities associated with arm, shoulder, and hand problems. The response options of DASH ranged from 1 to 5; 1 - no difficulty, 2 - mild difficulty, 3 - moderate difficulty, 4 - severe difficulty and 5 - unable. At least 27 out of 30 items were answered, then the score was calculated by summing up all responses and converted to a 0 to 100 scale. The scoring method for both FACT-B and DASH was done according to the instruction’s manual. In contrast to FACT-B score, a higher score in DASH reflects greater disability of the upper extremities. On the other hand, PHQ-ADS consists of the 9- item Patient Health Questionnaire (PHQ-9) and 7-item General Anxiety Disorder (GAD-7) and commonly used as anxiety and depression measures. Respondents were asked how much each symptom has bothered them for the past two weeks with response option of 0 to 3; 0 - not at all, 1 - several days, 2 - more than half the days and 3 - nearly every day. Just like DASH, the PHQ-9 and GAD-7 scores were calculated by summing up the responses and performed as continuous variables with higher scores representing more severe depression and anxiety (Kroenke et al., 2016). 

*Statistical analysis*

The internal consistency reliability of FACT-B was evaluated by calculating Cronbach’s alpha coefficient of each domain of FACT-B, FACT-G and TOI, with r ≥ 0.6 considered as an acceptable value (Sekaran, 2006). The findings were compared with other versions of FACT-B, including English, Korean and Malayalam. Concurrent validity was evaluated by calculating Pearson’s correlation coefficient (r) between all FACT-B domains (PWB, SWB, EWB, FWB, BCS) with DASH and PHQ-ADS scores. The r value of less than 0.30 indicates weak correlation; between 0.30 and 0.50 represents moderate correlation; above 0.5 is considered a strong correlation (Cohen, 1988). Meanwhile, known-group validity was carried out by dividing the subjects into two groups, without lymphedema (n = 83) and lymphedema (n = 30). The condition of having arm lymphedema was evaluated through two methods; which are self-reporting and arm circumference difference of more than 1.5 cm at two or more points of measurement; metacarpo-phalangeal junction, wrist, proximal forearm (10 cm away from elbow) and distal upper arm (15 cm from elbow) points. Descriptive statistics were analysed by comparing the mean difference scores and effect size of each of the FACT-B domains. Comparison of means between groups was done using student t-test and Mann-Whitney’s U test, with p < 0.01 or p < 0.05 as statistically significant and all statistical analysis were done using IBM SPSS Statistics version 25.0.

## Results

The mean age of participants during the recruitment was 50.1±8.8 years and the mean of years after diagnosis with breast cancer was 5.5±4.6 years. The distribution of baseline characteristics of all subjects is detailed in Table 1. 

*Reliability*

The response rate of all the domains were 100% except for SWB (98%) and BCS (98%). The mean score of FACT-B for all the participants was 116.0±17.9 and the Cronbach’s α value of each domain (PWB, SWB, EWB, FWB and BCS) ranged from 0.62 to 0.86. In addition, the Cronbach’s α value for TOI, FACT-G and FACT-B also showed excellent results (0.85 to 0.88) hence implying substantial reliability for the scale. The Cronbach’s α value of FACT-G and FACT-B were found to be in line with the validated English (Brady et al., 1997), Korean (Yoo et al., 2005) and Malayalam (Pandey et al., 2002) versions. Except for the BCS domain, moderate to high values were found in PWB, FWB, EWB and FWB across the different versions (Table 2). 

*Concurrent and known-group validity*

It is desirable to have data from concurrent administration of the instrument to be validated along with other questionnaires which measure the same concept (construct), a related concept, and an unrelated concept (Roberts and Priest, 2006). In Table 3, the r values showed the correlation not only between FACT-B domains but DASH and PHQ-ADS altogether. The total scores of FACT-B demonstrated strong correlations with both DASH (r = -0.613, p < 0.01) and PHQ-ADS (r = -0.610, p < 0.01) instruments. While all the FACT-B domains displayed significant correlation with DASH and PHQ-ADS (p < 0.01), the strength of the correlation varies between low and strong, depending on the items measured. For instance, DASH items are focused more on the physical and functional well-being measurement, which explains a strong correlation between PWB (r = -0.637, p < 0.01) with DASH. In contrast with DASH, PHQ-ADS showed moderate to strong correlations with EWB (r = -0.462, p < 0.01) and BCS (r = -0.541, p < 0.01) as compared to other domains, as both domains contained emotional and psychological related items. To assess the known-group validity of FACT-B, participants were divided into two groups, non-lymphedema (n = 83) and lymphedema (n = 30). As shown in Table 4, the scores of PWB, FWB, BCS and total scores of FACT-B between the group were significantly higher in the non-lymphedema group (p < 0.01), except for SWB and EWB. DASH score was found to be higher in the lymphedema group, with a large effect size of more than 0.5, indicating disability and movement of arms in affected individuals (p < 0.01). Meanwhile, no significant difference was observed between PHQ-ADS scores in both groups (p > 0.01). 

**Table 1:** Baseline Characteristic of the Study Population (n = 113)

Variables		Variables	
Age (years)	50.1±8.8	Income (RM), n	
BMI (kg/m^2^)	28.3±5.7	<3000	76
Time since diagnosed (years)	5.5±4.6	3000-8000	32
Marital status, n		>8000	5
Single	6	Stage of breast cancer, n	
Married	106	Stage I	22
Widowed	1	Stage II	58
Children, n		Stage III	26
Yes	94	Stage IV	7
No	19	Types of surgery, n	
Educational level		Lumpectomy	26
Primary & secondary school	67	Mastectomy	80
College & university	46	Lumpectomy & mastectomy	7
Occupation, n		Types of treatment, n	
Professional	21	Radiotherapy	86
Service/freelance	23	Chemotherapy	92
Homemaker/unemployed	60	Hormonal therapy	86
Retired	9	Arm lymphedema, n	30

Data were presented as mean with standard deviation (±SD) for age, body mass index (BMI) and years of survived.

**Table 2:** Comparison of Total Score and Internal Consistency (Cronbach’s alpha, α) of FACT-B with the Original (English) and Different Languages, Korean and Malayalam

Domains	Language							
	Malay (n = 113)		English (n = 295)		Korean (n = 201)		Malayalam (n = 31)	
	Mean (SD)	α	Mean (SD)	α	Mean (SD)	α	Mean (SD)	α
PWB (7 items)	22.0 (5.3)	0.85	22.1 (5.3)	0.81	21.3 (5.3)	0.81	19.1 (6.2)	0.75
SWB (7 items)	24.2 (5.0)	0.86	22.7 (5.2)	0.69	18.0 (4.5)	0.86	22.6 (5.2)	0.63
EWB (6 items)	19.4 (3.9)	0.67	16.3 (3.5)	0.69	16.4 (4.8)	0.79	15.5 (5.9)	0.84
FWB (7 items)	24.0 (4.5)	0.85	20.6 (6.4)	0.86	16.4 (5.6)	0.83	18.3 (6.8)	0.84
BCS (10 items)	26.3 (6.0)	0.62	24.1 (6.5)	0.63	21.3 (5.6)	0.67	18.5 (5.7)	0.41
TOI (24 items)	72.4 (12.7)	0.85	66.9 (15.3)	0.88	61.2 (13.6)	0.87	52.9 (11.5)	0.75
FACT-G (27 items)	89.7 (13.9)	0.88	88.8 (16.3)	0.9	71.2 (15.3)	0.87	75.9 (17.5)	0.9
FACT-B (37 items)	116.0 (17.9)	0.88	112.8 (20.9)	0.9	92.0 (22.6)	0.9	94.3 (22.1)	0.87

PWB, Physical well-being; SWB, social well-being; EWB, emotional well-being; FWB, functional well-being; BCS, breast cancer scale; TOI, total of index (PWB+FWB+BCS); FACT-G, is the total of PWB+SWB+EWB+FWB

**Table 3:** Correlation between FACT-B Domains with DASH and PHQ-ADS

Domains	PWB	SWB	EWB	FWB	BCS	FACT-B	DASH	PHQ-ADS
PWB	1	0.235^a^	0.323	0.471	0.459	0.707	-0.637	-0.477
SWB		1	0.348	0.577	0.266	0.659	-0.281	-0.268
EWB			1	0.426	0.491	0.682	-0.298	-0.462
FWB				1	0.454	0.797	-0.502	-0.441
BCS					1	0.767	-0.458	-0.541
FACT-B						1	-0.613	-0.610
DASH							1	0.368
PHQ-ADS								1

Pearson’s correlation coefficient All values are represented with significant p < 0.01, ^a^p value < 0.05

**Table 4:** Comparison of Mean Score and Effect Size of FACT-B Domains and DASH between Two Known-Groups

Domains	Without lymphedema (n=83)		Lymphedema (n=30)		p value	Effect size
	Mean	SD	Mean	SD		
PWB	23.2	4.3	18.7	6.6	0.001^b^	0.81
SWB	24.7	4.5	23.1	6.1	0.372^b^	0.29
EWB	19.7	3.6	18.5	4.4	0.204^b^	0.29
FWB	24.8	4.0	21.9	5.2	0.004^b^	0.63
BCS	27.1	6.0	24.1	5.5	0.015^a^	0.52
TOI	75.1	11.0	64.7	14.2	0.001^b^	0.82
FACT-G	92.5	10.7	82.1	17.9	0.010^b^	0.71
FACT-B	119.6	15.0	106.2	21.7	0.004^a^	0.72
DASH	17.2	12.6	35.4	20.2	<0.001^b^	1.08
PHQ-ADS	6.6	5.1	8.0	8.2	0.591^b^	0.21

^a^, Student t-test, p < 0.01; ^b^, Mann-Whitney U test, p < 0.05 SD: standard deviation

## Discussion

Assessment of QOL in cancer patients is important to reveal their coping mechanisms in all life aspects following cancer treatment. The two most used instruments in cancer research are Functional Assessment of Chronic Illness Therapy (FACIT) and European Organization for Research and Treatment of Cancer QOL Questionnaire (EORTC QLQ). Both instruments include core items that measure physical, functional, and psychosocial well-being of a cancer survivor, with the additional modules of items that are designed specifically for a particular cancer such as breast (EORTC QLQ BR23/FACT-B), colon (EORTC QLQ CR29/ FACT-C) or prostate cancer (EORTC-QLQ PR25/FACT-P). The Malay version of FACT-B was first translated in 2008 and the study was focused on factors affecting QOL in breast cancer patients (Redhwan et al., 2008). However, it is essential to validate a translated instrument as the reliability may be different under certain circumstances (i.e., population of the study). 

In the present study, a Cronbach’s α of FACT-B adapted in the Malay version showed a result of 0.88, indicating that the reliability is comparable to the original version, and in line with other adapted language versions. Excellent internal consistency was also found between PWB, SWB and FWB domains, whereas a low Cronbach’s α value was found in EWB (0.67) and BCS (0.62). One of the items in EWB, item GE2, ‘I am satisfied with how I am coping with my illness’ has been reported to be a misfit under emotional well-being, considering that the coping mechanism is associated with functional status (Porter et al., 2008). Interestingly, after removing item GE2, the EWB domain improved Cronbach’s α of 0.76 in our study. Considering that the time point after completed treatment of the participants are different (from three months to more than five years), we also postulate that different responses of participants on emotional well-being items might be influenced by the time of diagnosis and treatment endpoints (Turton and Cooke, 2000). Altogether, those who had just completed their treatment may have less self-efficacy the ability of oneself to face different consequences associated with cancer treatment or management (Foster et al., 2015). 

A low Cronbach’s α value in BCS was also reported in the original scale (Brady et al., 1997) and various FACT-B versions such as Korean (Yoo et al., 2005), Malayalam (Pandey et al., 2002) and Persian (Patoo et al., 2015). The variety of responses in this domain could be due to refusal of participants to answer sexual-related questions B4 (I feel sexually attractive) which was found to be at 98% of response rate. The response rate was also found to be only 91% for item GS7 (I am satisfied with my sex life) (data not shown). This finding is in line with a study that reported breast cancer women in a Malaysian cohort chose not to rate the questions about sexuality (Rajaram et al., 2019). Fewer responses on sexuality questions in FACT-B were also reported in Indian (Pandey et al., 2002), Arabic (Kobeissi et al., 2014) and Iranian (Patoo et al., 2015) cultures as open expression or discussion on sexuality is taboo in most Asian countries (Brotto et al., 2005; Rajaram et al., 2019). The heterogeneity of the two distinct groups in the study population; non-lymphedema and lymphedema, might also lead to the lower internal consistency value due to diverse responses in the BCS domain. Unlike the other general domains which have homogenous construct, BCS is uniquely designed to include important items identified in breast cancer, but not covered in the main domains, FACT-G (Brady et al., 1997). It is also important to note that BCS is only an additional scale in FACT-B and will never be used separately. Therefore, internal consistency may not be an appropriate measure as it works best for homogenous data (Yoo et al., 2005). Furthermore, when combined with other domains to produce TOI and FACT-B in the present study, the alpha coefficients were relatively high with 0.85 and 0.89, respectively. 

In the present study, we administered the Malay version of DASH and PHQ-ADS along with FACT-B to evaluate if they both measure similar physical, functional, and psychological distress status in the EWB or BCS domain. DASH is designed to measure upper extremity function and has been validated and used in the breast cancer population (Hayes et al., 2010; Harrington et al., 2011). The items enquire about the degree of difficulty in performing different physical activities and severity of pain associated with the arm, shoulder and hand. Our findings showed a moderate to strong correlation between DASH and all domains of FACT-B. A weak correlation was found between EWB and SWB with DASH implying that FACT-B has a distinct concept that can measure specific aspects in cancer patients. This is expected as DASH was not built primarily to assess mental health or psychosocial aspects, even though there were two or three items that maybe related to it, but not strong enough to correlate the findings with emotional or social well-being. Among the five domains, BCS displayed the highest correlation with the psychological-related instrument, PHQ-ADS that was applied in the study. A previous study by Yoo et al., (2005) reported that three factors were identified in the BCS scale after factor analysis, namely psychological distress, feminine satisfaction, and physical complaints. Therefore, the correlation of BCS domain with PHQ-ADS seems to be justified, considering that the BCS domain was not only measured the functional and physical well-being but the psychological condition too. 

Our known-group validity showed significantly higher scores of PWB, FWB, BCS, TOI, FACT-G and total FACT-B (p < 0.01), accompanied by lower DASH scores in the non-lymphedema group. This finding is in parallel with a previous validation study that suggested women with arm lymphedema had greater arm problems and significantly lower QOL (Beaulac et al., 2002). Besides PWB and FWB, BCS can be considered as reliable as it was able to assess the changes between two groups in the breast cancer population. Considering that the mean period after being diagnosed with breast cancer was 5.2 years, some of the participants may have started to face challenges from unwanted side effects following breast cancer treatment, such as arm lymphedema or sexual dysfunction (Bodai and Tuso, 2015). Furthermore, some items can be very specific to individuals with arm lymphedema, such as items B3 (One or both of my arms are swollen or tender) and P2 (I have certain parts of my body that I experienced pain). In contrast, the other important aspects like SWB and EWB were not comparable as no significant difference was found between the two groups. It has been suggested that emotional and social well-being are more stable and not dramatically changed over time when compared to physical and functional domains (Webster et al., 2003). Additionally, all participants involved in this study were recruited on a voluntary basis, from various cancer support groups. The informal support from peers who are or have been through similar health problems provide better psychological support as it can cushion the impact of stressors on an individual that makes them less vulnerable to unwanted consequences (Abu Kassim et al., 2015). Therefore, we can postulate that those who participated in the present study are socially stable due to social cohesion and encouraging members of their cancer support groups. 

In conclusion, our study demonstrated that psychometric properties in the Malay FACT-B are interconnected and reliably satisfactory to assess the QOL of Malaysian breast cancer patients, which is in line with the original (English) and other versions, such as Korean, Persian, and Indian. Therefore, we recommend the use of FACT-B in clinical research to assess the QOL for the Malaysian breast cancer population. 

## Author Contribution Statement

KMY wrote the manuscript, recruited the participants, analysed, and interpreted the participants’ data. MH co-supervised the study, K.A.A-K co-supervised and revised the manuscript, RM co-supervised, and validated the data of lymphedema patients. RR conceived, designed, and supervised the study, then revised and edited the manuscript. All authors read and approved the final manuscript.

## Data Availability

The datasets generated during the current study are not available publicly due to domestic regulation of the institution. However, they are available upon request to the corresponding author.

## References

[B1] Abu Kassim NL, Mohd Hanafiah K, Samad-Cheung H, Rahman MT (2015). Influence of support group intervention on quality of life of Malaysian breast cancer survivors. Asia Pac J Public Health.

[B2] Ahn E, Cho J, Shin DW (2009). Impact of breast cancer diagnosis and treatment on work-related life and factors affecting them. Breast Cancer Res Treat.

[B3] Arafat S, Chowdhury HR, Qusar MS, Hafez MA (2016). Cross Cultural Adaptation & Psychometric Validation of Research Instruments: a Methodological Review. J Behav Health.

[B4] Arifin W (2018). A web-based sample size calculator for reliability studies. Educ Med J.

[B5] Azamjah N, Soltan-Zadeh Y, Zayeri F (2019). Global trend of breast cancer mortality rate: A 25-Year Study. Asian Pac J Cancer Prev.

[B6] Beaulac SM, McNair LA, Scott TE, LaMorte WW, Kavanah MT (2002). Lymphedema and quality of life in survivors of early-stage breast cancer. Arch Surg.

[B7] Bodai BI, Tuso P (2015). Breast cancer survivorship: a comprehensive review of long-term medical issues and lifestyle recommendations. Perm J.

[B8] Brady MJ, Cella DF, Mo F (1997). Reliability and validity of the functional assessment of cancer therapy-breast quality-of-life instrument. J Clin Oncol.

[B9] Brotto LA, Chik HM, Ryder AG (2005). Acculturation and sexual function in Asian women. Arch Sex Behav.

[B10] Cheung YB, Luo N, Ng R (2014). Mapping the functional assessment of cancer therapy-breast (FACT-B) to the 5-level EuroQoL Group’s 5-dimension questionnaire (EQ-5D-5L) utility index in a multi-ethnic Asian population. Health Qual Life Outcomes.

[B12] Foster C, Breckons M, Cotterell P (2015). Cancer survivors’ self-efficacy to self-manage in the year following primary treatment. J Cancer Surviv.

[B13] Harrington S, Padua D, Battaglini C (2011). Comparison of shoulder flexibility, strength, and function between breast cancer survivors and healthy participants. J Cancer Surviv.

[B14] Hayes SC, Rye S, Battistutta D (2010). Prevalence of upper-body symptoms following breast cancer and its relationship with upper-body function and lymphedema. Lymphology.

[B15] Islam T, Bhoo-Pathy N, Su TT (2015). The Malaysian Breast Cancer Survivorship Cohort (MyBCC): a study protocol. BMJ Open.

[B16] Kobeissi L, Saad MA, Doumit M (2014). Face validity of the Functional Assessment of Cancer Therapy-Breast Symptom Index (FACT- B) into Formal Arabic. MEJC.

[B17] Kroenke K, Wu J, Yu Z (2016). Patient health questionnaire anxiety and depression scale: Initial Validation in Three Clinical Trials. Psychosom Med.

[B18] Martinez RB, Boronat OG, Badia MS (2011). Functional assessment of cancer therapy questionnaire for breast cancer (FACT-B+4). Spanish version validation. Med Clin (Barc).

[B19] Pandey M, Thomas BC, Ramdas K, Eremenco S, Nair MK (2002). Quality of life in breast cancer patients: validation of a FACT-B Malayalam version. Qual Life Res.

[B20] Patoo M, Allahyari AA, Moradi AR, Payandeh M (2015). Persian version of functional assessment of cancer therapy- Breast (FACT-B) Scale: Confirmatory Factor Analysis and Psychometric Properties. Asian Pac J Cancer Prev.

[B21] Porter LS, Keefe FJ, Garst J, McBride CM, Baucom D (2008). Self-efficacy for managing pain, symptoms, and function in patients with lung cancer and their informal caregivers: associations with symptoms and distress. Pain.

[B22] Rajaram N, Lim ZY, Song CV (2019). Patient-reported outcome measures among breast cancer survivors: A cross-sectional comparison between Malaysia and high-income countries. Psychooncology.

[B23] Redhwan AA, Idris M, Zaleha M (2008). Quality of life among women with breast cancer from Universiti Kebangsaan Malaysia Medical Centre, Malaysia. J Comm Health.

[B24] Roberts P, Priest H (2006). Reliability and validity in research. Nurs Stand.

[B26] Shamsudin ID, Yu ML, Brown T (2018). Translation, cross-cultural adaptation and validation of the Quality of Life in Autism Questionnaire (QoLA) from English into the Malay language. J Occup Ther Sch Early Interv.

[B27] Sherina MS, Arroll B, Goodyear-Smith F (2012). Criterion validity of the PHQ-9 (Malay version) in a primary care clinic in Malaysia. Med J Malaysia.

[B28] Sidik SM, Arroll B, Smith GF (2012). Validation of the GAD-7 (Malay version) among women attending a primary care clinic in Malaysia. J Prim Health Care.

[B29] Turton P, Cooke H (2000). Meeting the needs of people with cancer for support and self-management. Complement Ther Nurs Midwifery.

[B30] Webster K, Cella D, Yost K (2003). The Functional Assessment of Chronic Illness Therapy (FACIT) Measurement System: properties, applications, and interpretation. Health Qual Life Outcomes.

[B31] Yoo HJ, Ahn SH, Eremenco S (2005). Korean translation and validation of the functional assessment of cancer therapy-breast (FACT-B) scale version 4. Qual Life Res.

